# Sex hormones influence *ORMDL3* expression: Implications for sex-associated asthma phenotype

**DOI:** 10.1016/j.jaci.2025.10.035

**Published:** 2025-12-06

**Authors:** Elisabetta Granato, Ida Cerqua, Antonietta Rossi, Maria Antonietta Riemma, Maria Chiara Monti, Giusy Ferraro, Barbara Romano, Maria Francesca Nanı, Danilo D’Avino, Martina Simonelli, Joshua Malo, Francesca Polverino, Bruno D’Agostino, Giuseppe Cirino, Fiorentina Roviezzo

**Affiliations:** a Department of Pharmacy, School of Medicine and Surgery; b GENESIS–Interdepartmental Research Centre in Gender Medicine, University of Naples Federico II, Naples; c Department of Environmental Biological and Pharmaceutical Sciences and Technologies, University of Campania Luigi Vanvitelli, Naples; d Department of Medicine, Pulmonary and Critical Care Medicine, Baylor College of Medicine, Neurosensory Center of Houston, Houston; e Department of Pulmonary and Critical Care Medicine, University of Arizona, Tucson

**Keywords:** ORMDL3, sphingolipid metabolism, airway function, asthma, sex

## Abstract

**Background:**

Genetic polymorphisms in sphingolipid metabolism, particularly involving the orosomucoid-like 3 *(ORMDL3)* gene, have been associated with asthma risk. Notably, asthma prevalence and severity exhibit pronounced sex differences, emerging in childhood and persisting into adulthood. However, the molecular mechanisms underlying this sexual dimorphism remain incompletely elucidated.

**Objective:**

We investigated whether *ORMDL3* contributes to sex differences in airway function and asthma-like features.

**Methods:**

*ORMDL3* expression was measured in lung tissues from healthy male and female human donors and in human bronchial epithelial cells (BEAS-2B) after exposure to 17β-estradiol (E2), *Dermatophagoides pteronyssinus* 1 (Der p 1), or both. A murine preparation of asthma was used to evaluate sex-dependent differences in *ORMDL3* expression, airway responsiveness, and remodeling. Pharmacologic modulation of estrogen signaling and the ORMDL3–sphingosine-1-phosphate (S1P) axis were used. Methods included quantitative real-time PCR, immunostaining, liquid chromatography–tandem mass spectrometry, and airway function measurements.

**Results:**

Female lungs exhibited higher *ORMDL3* expression than male lungs, and this correlated with elevated forced expiratory volume to forced vital capacity ratios in the same patients. In BEAS-2B cells, E2 significantly upregulated ORMDL3 and altered sphingolipid metabolism by inducing expression of ceramidase, sphingosine kinases 1/2, and S1P receptors. Der p 1 also increased ORMDL3 and triggered epithelial activation via inflammasome signaling, while E2 enhanced IFN-β signaling and *MUC5AC* expression. Combination Der p 1/E2 synergistically activated sphingolipid, interferon, and inflammasome pathways. These *in vitro* findings prompted *in vivo* investigation using a murine asthma preparation, where female mice displayed elevated ORMDL3, sphingosine, and S1P levels, with increased airway hyperresponsiveness and remodeling. Treatment with tamoxifen or E2 normalized airway hyperresponsiveness and S1P signaling across sexes. Allergen sensitization intensified female-biased *ORMDL3* expression and airway inflammation. Inhibition of the ORMDL3-S1P axis attenuated asthma-like features only in female animals.

**Conclusion:**

This study identifies ORMDL3 as an estrogen-responsive regulator of airway responsiveness that may contribute to sex-related differences in asthma features through modulation of sphingolipid metabolism.

## Capsule summary

*ORMDL3* expression is regulated in a sex-dependent manner, influenced by estradiol and associated with alterations in sphingolipid signaling. These findings support the concept of hormone-responsive asthma endotypes and may inform future personalized treatment strategies targeting lipid metabolism pathways.

## Graphical Abstract

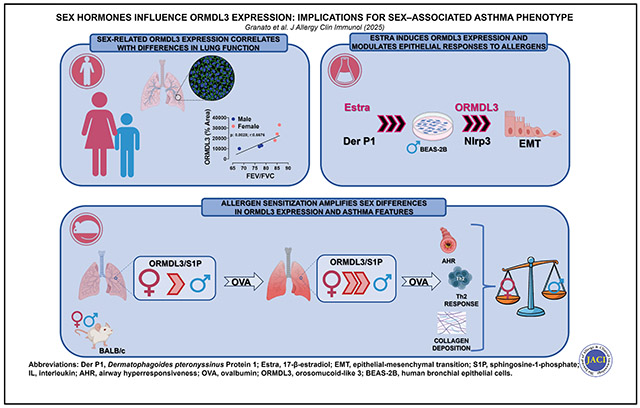

Asthma is a chronic inflammatory airway disease influenced by genetic, environmental, and physiologic factors, with pronounced sexual dimorphism in its prevalence, severity, and clinical manifestation.^1,2^ Orosomucoid-like 3 (ORMDL3) is an endoplasmic reticulum–localized transmembrane protein that plays a central role in sphingolipid metabolism by negatively regulating serine palmitoyl–coenzyme A transferase (SPT), the rate-limiting enzyme in *de novo* sphingolipid biosynthesis.^3^ Sphingolipids such as ceramides and sphingosine-1-phosphate (S1P) are critical for maintaining membrane structure, intracellular signaling, and coordinating inflammatory responses, making their homeostatic regulation essential.^4,5^ ORMDL3-mediated inhibition of SPT serves as a key checkpoint in preventing sphingolipid dysregulation.^6^ Elevated ceramide and S1P levels have been implicated in asthma pathogenesis, contributing to airway inflammation, bronchial hyperresponsiveness, and immune cell recruitment.^7,8^ Although ORMDL3 is recognized as a physiologic suppressor of ceramide biosynthesis, the regulatory mechanisms governing *ORMDL3* expression in inflammatory contexts, particularly in allergic asthma, remain incompletely understood. The balance between ceramide and S1P, regulated by a network of enzymes including sphingomyelinases, ceramidases, sphingosine kinases (SphK1/2), transporter proteins (eg, Spns2), and S1P lyase, is instrumental in determining cellular responses such as survival, apoptosis, and inflammation.^9-11^ Genome-wide association studies have robustly linked *ORMDL3* variants to increased risk for childhood-onset asthma, a finding that has been consistently validated across diverse populations.^12^

Beyond genetic susceptibility, sex-based differences in asthma pathophysiology are increasingly attributed to hormonal and immunomodulatory factors.^1^ While asthma is more prevalent in boys before puberty, a reversal occurs after puberty, with adult women exhibiting a higher disease burden.^2,13^ Fluctuations in asthma severity during reproductive milestones (eg, menstrual cycle, pregnancy, menopause) further underscore the influence of sex hormones, particularly estrogen, on airway inflammation and responsiveness. Women are more likely to report severe symptoms and a greater reliance on bronchodilators, potentially as a result of estrogen-mediated modulation of inflammatory and metabolic pathways.^14^ Estrogen signaling operates through both genomic and nongenomic mechanisms, including cross talk with sphingolipid metabolism. Notably, circulating S1P levels are significantly higher in female subjects and positively correlate with estrogen concentrations, implicating hormonal control of sphingolipid biosynthesis.^15^ Moreover, sex-specific genetic associations have emerged, with certain *ORMDL3* single nucleotide polymorphisms demonstrating stronger correlations with asthma risk in female subjects.^16^

These observations reveal a complex interplay among sex hormones, sphingolipid metabolism, and genetic regulation that likely contributes to sex disparities in asthma. In this study, we identify the ORMDL3-S1P signaling axis as a sex-regulated molecular pathway underlying differential airway responses between male and female subjects. Our findings suggest that estrogen-driven upregulation of ORMDL3 and its downstream effects on sphingolipid metabolism may shape sex-specific asthma susceptibility. This pathway appears to influence key features of asthma pathology, including airway hyperresponsiveness (AHR), immune activation, and structural remodeling, offering novel mechanistic insight and a potential framework for the development of sex-tailored therapeutic strategies.

## METHODS

### Human studies

All study protocols and patients’ written consent forms were approved by the institutional review board of the University of Arizona (no. 1811124026) in accordance with the Declaration of Helsinki. The human lung tissue sections used in this study, obtained from both male and female donors (n = 9), were control samples sourced from patients without chronic obstructive pulmonary disease or other underlying primary lung diseases. These control samples served as a baseline for comparison with chronic obstructive pulmonary disease patient tissues and were provided by Dr Polverino’s lung biobank. Lung tissue sections from both male and female never-smoker control subjects (n = 9) were obtained from archived specimens collected during lung transplantation or wedge resections/lobectomies performed for clinical purposes. Lung tissue sections were used for immunofluorescence (IF) analysis. Patient demographic information, comorbidities, and lung function parameters are provided in Tables E1 and E2 in this article’s Online Repository available at www.jacionline.org.

### Cell culture and treatments

Male human bronchial epithelial cells (BEAS-2B) were obtained from the American Type Culture Collection (ATCC, Manassas, Va) and cultured in 75 cm^2^ flasks at a density of 1 × 10^6^ cells. Cells were maintained at 37°C in a humidified incubator with 5% CO_2_, and the culture medium was refreshed every 48 hours until 90% confluence was reached. For hormone stimulation, cells were treated with 17β-estradiol (E2; 0.25 μmol; Sigma-Aldrich, St Louis, Mo, E4389) or vehicle every other day for 5 days. Subsequently cells were exposed to Der p 1 (10 ng/mL; MyBioSource, Vancouver, British Columbia, Canada, MBS485103) for 24 hours. At the conclusion of each treatment, cells were collected for downstream analyses, including real-time quantitative PCR (qPCR) and IF.

### Animal studies

Adult female and male BALB/c mice (8-9 weeks old) were obtained from Charles River Laboratories (Calco, Italy). Animals were housed under specific-pathogen–free conditions in a controlled environment with a 12-hour light/dark cycle and had free access to water. Male mice received a daily subcutaneous injection of E2 (25 μg/kg; Sigma-Aldrich, E8875) or vehicle for 1 week. Female mice received a daily subcutaneous injection of tamoxifen, an estrogen receptor antagonist (0.4 mg/kg; Sigma-Aldrich, T5648), or vehicle for the same duration. Mice were humanely killed 7 days after the first injection. At the time of death, animals were anesthetized and humanely killed according to approved protocols. Blood, main bronchi, and lungs were collected for molecular and functional analyses. The lungs were divided as follows: the right lobes were snap frozen and stored at −80°C for later homogenization and analysis of cytokine and sphingolipid content, while the left lobes were fixed in 4% buffered formalin for histologic and immunohistochemical evaluation.^17^ Main bronchi were isolated and processed for functional studies, Western blot analysis, and qPCR analysis.

All experiments were conducted during daylight according to Italian regulations on the protection of animals used for experiments and other scientific purposes (D.Lgs. 26/2014) as well as with the European Economic Community regulations (EU Directive 2010/63/EU). Animal studies are reported in compliance with the ARRIVE guidelines.

### Experimental model of asthma

Six each of male and female BALB/c mice received subcutaneous injections of chicken egg white ovalbumin (OVA; 100 μg, grade V, Sigma-Aldrich, A5503) emulsified in 3.3 mg of aluminum hydroxide gel (Al(OH)_3_; Supelco, Bellefonte, Pa, 101091) in sterile saline (400 μL) on days 0 and 7. Control animals received an equivalent volume of sterile saline. Mice were humanely killed 21 days after the first OVA administration for sample collection.^18^ In a separate experimental group, mice were treated intraperitoneally with l-cycloserine, a serine palmitoyltransferase (SPT) inhibitor (100 mg/kg; 100 μL), administered 30 minutes before each OVA injection. At the end point (day 21), all animals were anesthetized and humanely killed. Blood, main bronchi, and lungs were collected for functional and molecular analyses. The left lung lobes were fixed in 4% buffered formalin for histologic and immunohistochemical evaluation, while main bronchi were isolated for airway reactivity studies.

### Airway responsiveness measurements

Bronchial rings 2 mm long were collected from BALB/c mice and transferred in organ baths (3 mL) filled with Krebs solution at 37°C and oxygenated with a mix of 95% O2 and 5% CO2. In each experiment, the bronchial rings were previously challenged with carbachol (10^−6^ mol) until the responses were reproducible. Once a reproducible response was obtained, bronchial reactivity was tested by performing a cumulative concentration–response curve to carbachol (10^−6^ mol to 10^−6^ mol). In another set of experiments, the main bronchi were incubated with JTE-013 (10 μmol, 15 minutes), a S1PR2 antagonist and with SKI-II (100 μmol, 1 hour), a SphK inhibitor After incubation, the bronchi were stimulated with a cumulative concentration– response curve of carbachol as previously described.^18^ Data were expressed as contraction capacity compared to increasing concentration of carbachol (dyne/mg of tissue).

### Measurement of plasma IgE and cytokine levels

The pulmonary levels of IL-5 (Affymetrix, eBioscience, San Diego, Calif) and the plasma IgE levels (BD Biosciences, San Jose, Calif) were measured with commercially available ELISA kits according to the manufacturer’s instructions. Levels of cytokines were expressed as picograms per milligram of tissue.^19^

### S1P dosage

Extraction of sphingosine (Sph) and S1P from homogenates of lung tissue was performed by the following method.^20^ Briefly, 1 mL of mixture *i*–PrOH–water–EtOAc (30:10:60) was added to 0.5 mg of total proteins measured by Bradford assay in each lung tissue homogenate. Samples were stirred for 30 minutes, sonicated for 15 minutes, and centrifuged at 21,000 3 *g* for 15 minutes. The supernatant was transferred to a new centrifuge tube and evaporated to dryness at 40°C in the vacuum rotator. Samples were reconstituted in 50 μL MeOH, vortexed for 5 minutes, sonicated for 10 minutes, and centrifuged for 10 minutes at 10,000 × *g* and 10 μL were loaded on the high-performance liquid chromatography–tandem mass spectrometry (HPLC-MS/MS) system. Sph and S1P were quantified by HPLC-MS/MS on Shimadzu LC-20A and Auto Sampler systems and QTRAP 6500 instrument from AB-Sciex. C18 chromatographic column (Kinetex C18, 50 × 2.1 mm, 5 μm, Phenomenex, Torrance, Calif) was used for chromatographic separation. The mobile phase was composed of water 0.1% formic acid (mobile phase A) and methanol 0.1% formic acid (mobile phase B). The flow rate was set at 400 μL/min. The mass spectrometer was set in the positive ion mode (ESI^1^ ) with an electrospray voltage of 5500 V at 400°C of the heated capillary temperature. Multiple reaction monitoring mode and Analyst 1.6.2 software were used. Sph was analyzed with the mass transition 300 *m/z* to 282 *m/z,* and it was observed at the retention time (rt) of 7.18 minutes. S1P was analyzed with the mass transition 380 *m/z* to 264 *m/z* and it was observed at the rt of 8.30 minutes. Nitrogen was used as the air curtain gas (20 psi), atomizing gas (30 psi), auxiliary gas (60 psi), and collision gas (4 psi). Dwell time was 100 ms, DP was 74v, EP was 10v, CE was 22v, and CXP was 15v. For quantitative analysis, a standard curve with Sph and S1P amounts of 250 pg, 100 pg, 25 pg, 10 pg, and 2.5 pg was run.

### Lung histology, immunohistochemistry, and IF

Human and mouse lung sections (7 μm thick) were mounted on glass slides, deparaffinized, and rehydrated using standard protocols. For immunohistochemistry (IHC) analysis, endogenous peroxidase activity was quenched with 3% hydrogen peroxide for 15 minutes at room temperature, followed by blocking in 3% bovine serum albumin (Sigma-Aldrich, A9418) to reduce nonspecific binding. Slides were incubated overnight at 4°C with anti-SPNS2 antibody (1:100, Abcam, Cambridge, England, United Kingdom, ab82629). The next day, sections were incubated with a peroxidase-conjugated goat anti-rabbit secondary antibody (1:500, Jackson ImmunoResearch Laboratories, West Grove, Pa, 111-035-144). Immunoreactivity was visualized using SIGMAFAST 3,3’-diaminobenzidine (aka DAB) chromogen. Negative controls were processed in parallel using only the secondary antibody. Images were acquired using a Leica microscope at 40× magnification (Leica, Wetzlar, Germany). For IF, sections were incubated overnight at 4°C with anti-ORMDL3 (1:100, Abcam, ab211522) and anti–α-smooth muscle actin (α-SMA; 1:100, Sigma-Aldrich, A5228). The next day, slides were incubated for 1 hour at room temperature with Alexa Fluor 488–conjugated goat anti-mouse (1:1000, Invitrogen; Thermo Fisher Scientific, Waltham, Mass, A28175) or Alexa Fluor 568–conjugated goat anti-rabbit (1:1000, Invitrogen, A-11012) secondary antibodies. Negative controls omitted primary antibodies. Images were captured with a Zeiss confocal microscope and ZEN 3.2 software (Carl Zeiss, Jena, Germany). Additional sections were stained with hematoxylin (8 minutes) and eosin (seconds) for structural assessment.^21^ Lung inflammation was semiquantitatively scored using the 0-to-5 Ashcroft scale, where 0 represents normal tissue and 5 indicates severe inflammation. A score of 1 corresponds to minimal epithelial damage, minimal alveolar septal thickening, and minimal eosinophil infiltration; scores from 2 to 5 reflect a progressive increase in all the characteristics. Semiquantitative analysis of IHC and IF staining was performed by ImageJ/Fiji software (imagej.net/ij) to assess relative protein expression.

### Flow cytometry

Fresh lungs were excised in sterile conditions and minced in digestion buffer containing a mixture of collagenase D (1 mg/mL, Roche, Basel, Switzerland, 11088858001) and DNase I (0.1 mg/mL, Roche, 10104159001) in Hanks balanced salt solution with 5% fetal bovine serum. Tissue was digested with continuous agitation of 250 rpm at 37°C. After 30 minutes of incubation and agitation, the cell suspension was then passed through a 70 μm cell strainer and centrifuged at 1300 rpm for 10 minutes at room temperature. Red blood cells were removed with RBC Lysis buffer (Sigma-Aldrich, R7757), washed with fluorescence-activated cell sorting buffer, and then centrifuged again at 1300 rpm for 10 minutes. Approximately 1 × 10^6^ cells per sample were incubated with FcBlock (BD Biosciences), then stained with a mixture of fluorochrome-conjugated antibodies as follows: (1) CD11c-APC, cKit-PeCy5.5, IgE–fluorescein isothiocyanate (Bioscience, San Diego, Calif) for mast cells; and (2) anti–CD4-Pe, anti–CD45R-APC antibodies (eBioscience). Events (2 × 10^4^) from lung cell suspensions were acquired with a BD FACSCalibur (BD Biosciences). Data analysis was performed by FlowJo software (Becton Dickinson, Franklin Lakes, NJ).

### RNA extraction and gene expression profiling by qPCR

Mouse and cellular samples were homogenized by FastPrep in PureZOL reagent (Bio-Rad, Hercules, Calif) and RNA was extracted according to manufacturer’s instructions. Quantification and quality analysis of RNA were ascertained by a NanoDrop One/OneC Microvolume UV-Vis spectrophotometer (Thermo Fisher Scientific). Retrotranscription was performed by using the High-Capacity cDNA Reverse Transcription Kit (Applied Biosystems; Thermo Fisher Scientific). qPCR was performed by using the Fast SYBR Green Master Mix (Applied Biosystems), and reactions were performed and analyzed on CFX Connect Real-Time PCR Detection System (Bio-Rad). Target gene expression calculations were normalized on reference housekeeping gene *GAPDH* (glyceraldehyde-3-phosphate dehydrogenase) and expressed by using the 2^−ΔCt^ (for mouse samples) or 2^−ΔΔCt^ (for cellular samples) formula. Primers used are summarized in Table E3 in the Online Repository available at www.jacionline.org.

### Western blot analyses

Mouse bronchi were homogenized in radioimmunoprecipitation assay lysis buffer supplemented with a protease inhibitor cocktail (Sigma-Aldrich, P8340). Total protein (40 μg) was separated by SDS-PAGE and transferred onto a polyvinyl difluoride membrane. After blocking with 5% nonfat milk in PBS-T (PBS + 0.1% Tween 20), membranes were incubated overnight at 4°C with a primary antibody against ASAH1 (1:1000, Elabscience, Houston, Tex, E-AB-10959). After washing in PBS-T, membranes were incubated with a peroxidase-conjugated goat anti-rabbit secondary antibody (1:500, Jackson ImmunoResearch, 111-035-144) for 2 hours at room temperature. Protein bands were visualized using Clarity Western enhanced chemiluminescence substrate (Bio-Rad) and detected with a ChemiDoc imaging system. Densitometric analysis was performed with an ImageQuant 400 device (GE Healthcare, Waukesha, Wis). *ASAH1* expression was normalized to the housekeeping protein GAPDH (1:5000, Invitrogen) to account for loading variability.

### Statistical analysis

The results were expressed as means ± SEMs of the mean of observations, where n represents the number of animals or the number of experiments performed on different days. In the experiments involving histology, the figures shown are representative of 6 animals. Statistical evaluation was performed by unpaired *t* test or 1-way or 2-way ANOVA followed by Bonferroni as a *post hoc* test by GraphPad Instant software (GraphPad Software, La Jolla, Calif). A significance threshold of *P* <.05 was used.

## RESULTS

### *ORMDL3* expression in lung is modulated by sex

**FIG 1.**
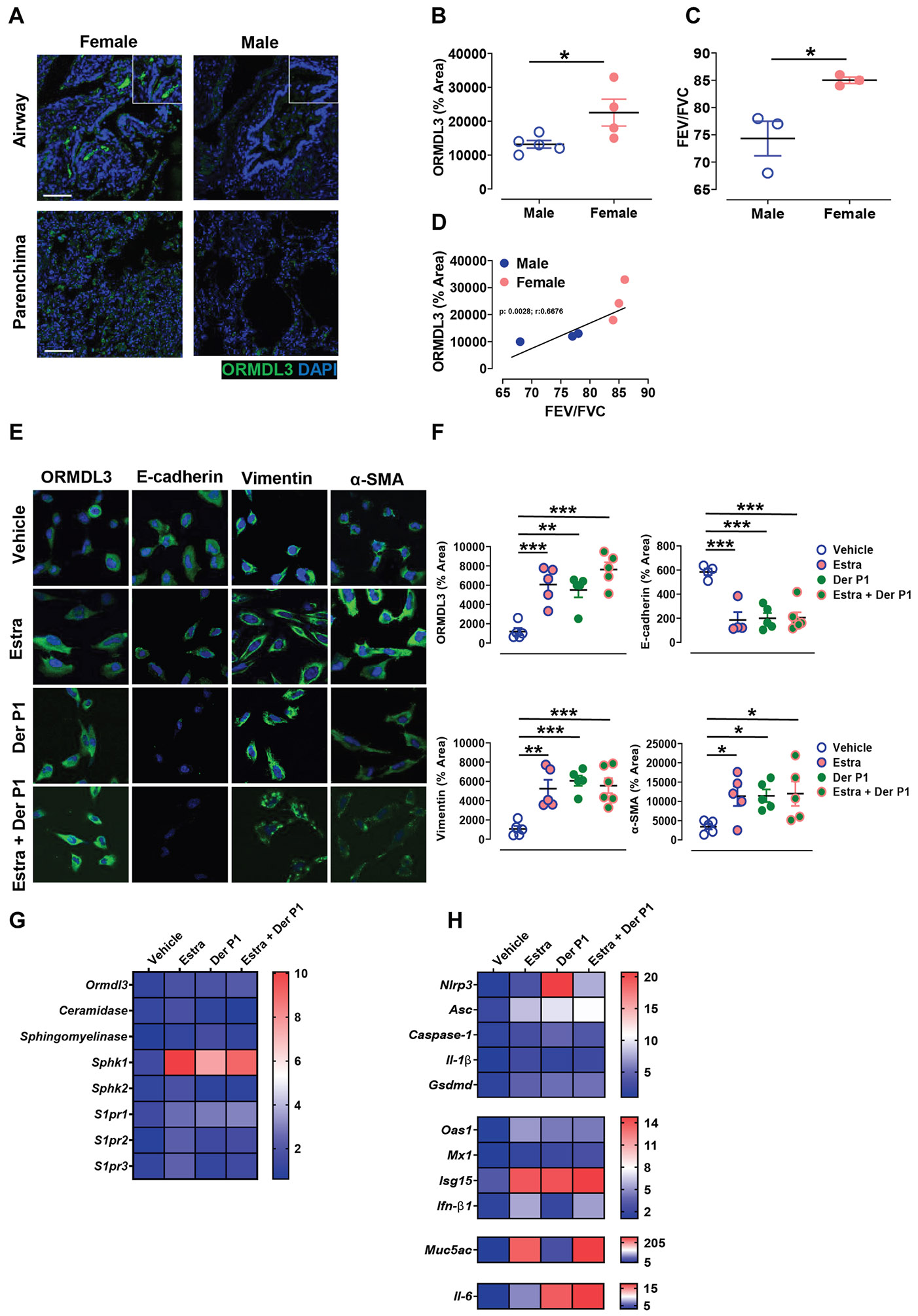
*ORMDL3* expression in lung is modulated by sex. **(A)** Representative IF staining for ORMDL3 in human lung tissue sections, obtained from healthy male and female donors (scale bar = 169.6 μm). **(B)** Semiquantitative analysis by ImageJ/Fiji for ORMDL3. **(C)** FEV_1_/FVC in healthy male and female subjects. **(D)** Correlation between ORMDL3 bronchial staining intensity and FEV_1_/FVC in male and female subjects. **(E)** Representative IF staining in BEAS-2B cells treated with vehicle, E2 (Estra, 0.25 μmol), Der p 1 (10 ng/mL), or both (scale bar = 85.6 μm). **(F)** Semiquantitative analysis of ORMDL3, E-cadherin, vimentin, and α-SMA expression by ImageJ/Fiji software. **(G)** Heat map of qPCR (2^−ΔΔ*Ct*^) values for *ORMDL3, Ceramidase, Sphingomyelinase, SPHK1, SPHK2, S1PR1, S1PR2*, and *S1PR3* in BEAS-2B cells. **(H)** Heat map of qPCR (2^−ΔΔ*Ct*^) values for genes involved in inflammasome pathway (*NLRP3, ASC, Caspase-1, IL-1β, GSDMD*), type I interferon pathway (*OAS1, MX1, ISG15, IFNB1*), and mucus/inflammatory markers (*MUC5AC, IL6*). Data are expressed as means ± SEMs. Statistical analysis: unpaired *t* test (*B* and *C*), Spearman correlation test *(D)*, 1-way ANOVA *(F)*. *P < .05, **P < .01, ***P < .001 vs vehicle.

Lung sections from surgical resections or explants were obtained from male and female donors without chronic lung diseases (4 female and 5 male subjects; Tables E1 and E2). IF staining was performed to assess *ORMDL3* expression (Fig 1, A). *ORMDL3* was more expressed in female lung tissue compared to male tissue (Fig 1, A and B), with positive immunoreactivity predominantly localized to the bronchial epithelial layer.

This sex-related difference in *ORMDL3* expression was accompanied by a corresponding difference in the ratio of forced expiratory volume in 1 second (FEV_1_) to forced vital capacity (FVC), a key index of bronchial function used to assess airway obstruction or restriction. Female donors showed higher FEV_1_/FVC ratios than male donors while remaining within the normal range (Tables E1 and E2). This may reflect sex-based differences in airway structure or in genetic expression patterns such as *ORMDL3* (Fig 1, C). Despite the limited sample size, the difference in FEV_1_/FVC between sexes was statistically significant. Furthermore, a strong positive correlation was observed between *ORMDL3* expression and FEV_1_/FVC (Fig 1, D), suggesting that higher *ORMDL3* expression is associated with higher airway patency as estimated by the FEV_1_/FVC (%) ratio.

### E2 induces *ORMDL3* expression and modulates epithelial responses to allergens

For hormone stimulation, male epithelial cells such as BEAS-2B were treated with E2 (0.25 μmol) or vehicle every other day for 5 days. Cells were subsequently exposed to Der p 1. We compared the responses of BEAS-2B cells exposed to Der p 1 or E2 and their combination. Treatment with either E2 or Der p 1 resulted in *ORMDL3* upregulation (Fig 1, E and F, and see Fig E1 in the Online Repository available at www.jacionline.org). Both stimuli induced cell activation as demonstrated by epithelial–mesenchymal transition (Fig 1, E and F) but engaged distinct signaling pathways and effectors (Fig 1, E-H). Notably, Der p 1 exposure did not significantly alter sphingolipid metabolism, whereas E2 caused marked changes in this pathway (Fig 1, G, and Fig E1). Genes encoding key enzymes involved in sphingolipid metabolism, including ceramidase, SphK1, and SphK2, as well as S1P receptors S1PR1, S1PR2, and S1PR3, showed a significant increased expression in E2-stimulated cells (Fig 1, G, and Fig E1). Der p 1 exposure predominantly triggers epithelial activation associated with inflammasome activation (Fig 1, G and H*,* and Fig E1), while E2 robustly promotes IFN-β signaling and enhances S1P pathway activation (Fig 1, G and H, and Fig E1). In addition, E2 significantly upregulated MUC5AC (Fig 1, H, and Fig E1), while Der p 1 more significantly upregulated IL-6 (Fig 1, H, and Fig E1). Moreover, coexposure to both Der p 1 and E2 results in a synergistic activation that integrates inflammasome, interferon, and sphingolipid signaling pathways, thereby recapitulating the full spectrum of molecular responses observed individually (Fig 1, E-H, and Fig E1).

### Sex differences in *ORMDL3* expression correlate with AHR and airway S1P signaling in BALB/c mice

**FIG 2.**
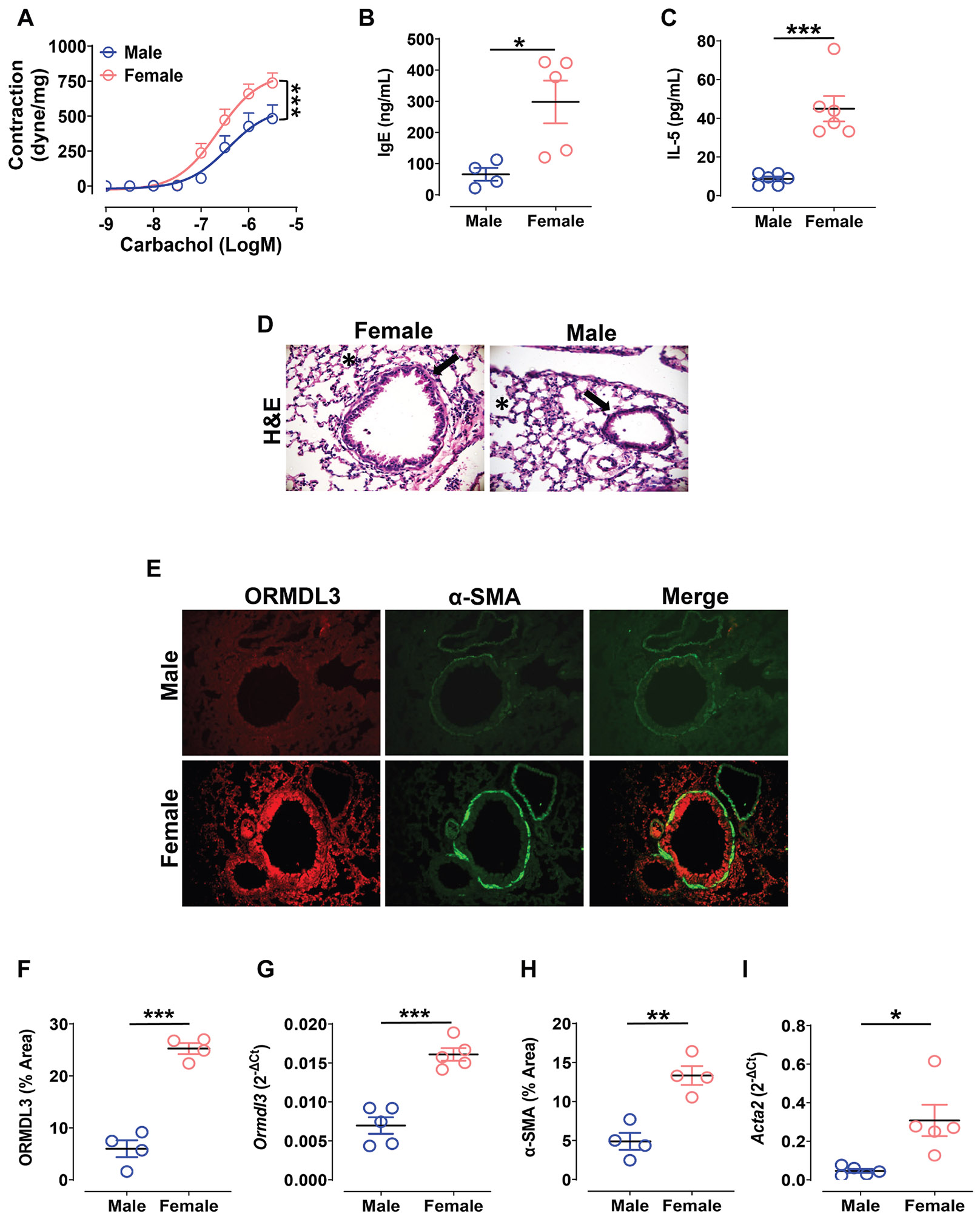
Sex affects airway *ORMDL3* expression and function in BALB/c mice. **(A)** Airway reactivity in isolated bronchi from male and female naïve BALB/c mice. **(B)** Plasma IgE levels. **(C)** Pulmonary IL-5 expression measured by ELISA. **(D)** Representative hematoxylin and eosin staining of lung sections (scale bar = 169.6 μm). **(E)** IF staining for ORMDL3 and α-SMA in lung sections (scale bar = 169.6 μm). (**F** and **H**) Semiquantitative analysis by ImageJ/Fiji for ORMDL3 and α-SMA. (**G** and **I**) qPCR quantification of *Ormdl3* and *Acta2* (α-SMA gene). Data are expressed as means ± SEMs. *(F)* *P < .05, **P < .01, ***P < .001. Statistical analysis: 2-way ANOVA with Bonferroni posttest *(A)*, unpaired *t* test (*B, C, F, G, H*, and *I*). *P < .05, **P < .01. ***P < .001.

BALB/c mice are a T_H_2 immunophenotype strain sensitive to allergen sensitization with higher susceptibility in female animals.^22^ In line with this, we found that bronchi collected from female mice displayed a higher reactivity to carbachol compared to male mice (Fig 2, A). The higher reactivity to carbachol correlated with basal higher plasma levels of IgE (Fig 2, B) and higher pulmonary expression of IL-5 in female compared to male animals (Fig 2, C). Further, hematoxylin and eosin staining showed that naïve male and female mice had differences in lung structure and in the alveolar septa, with larger septa in male mice (Fig 2, D*, asterisk*) and a thicker smooth muscle layer in female mice (Fig 2, D*, arrow*).

**FIG 3.**
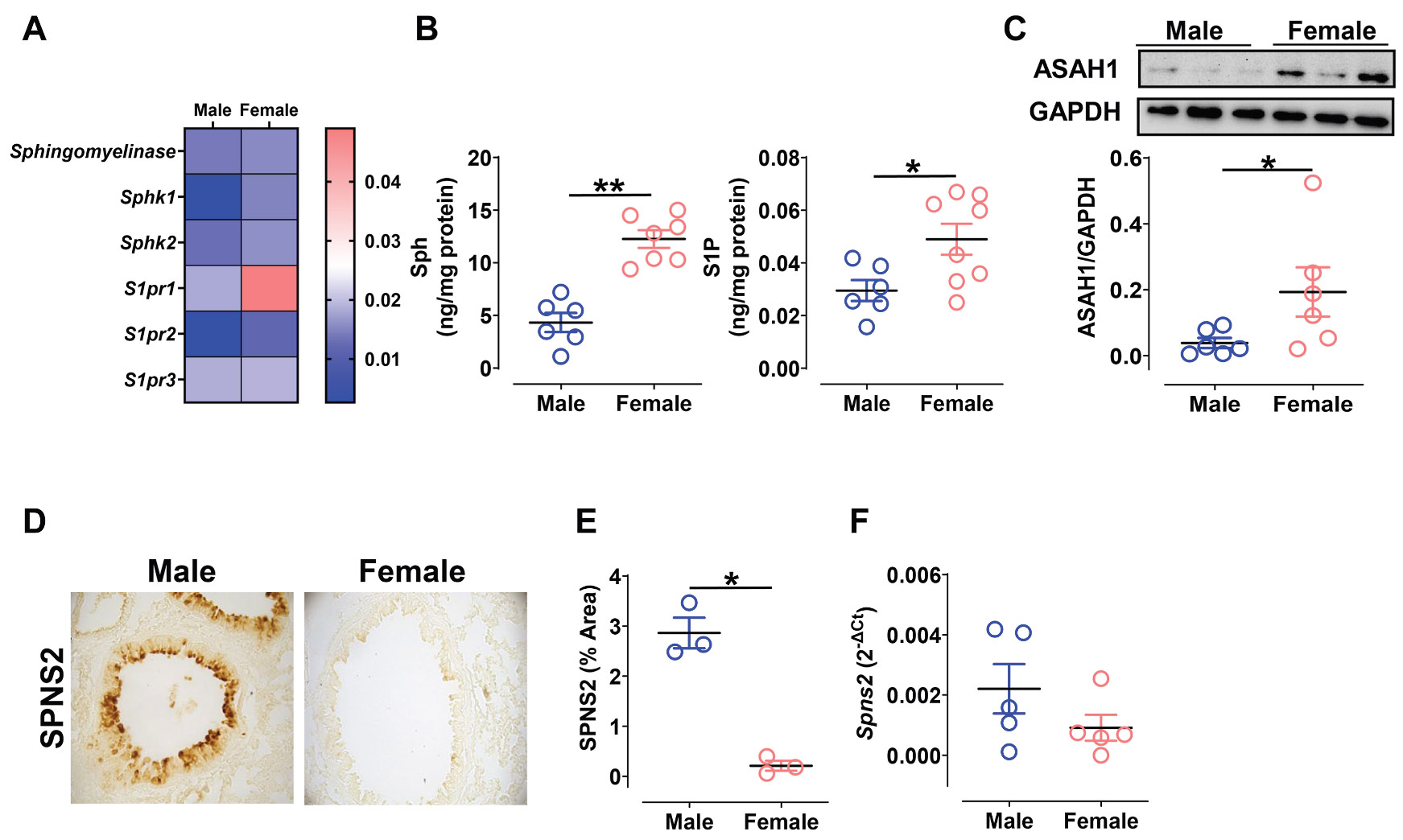
S1P signaling is upregulated in female mice. Lung and bronchial tissues collected from male and female naïve BALB/c mice were used for: **(A)** heat map of qPCR (2^−Δ*Ct*^) for *Sphingomyelinase, Sphk1, Sphk2, S1pr1, S1pr2*, and *S1pr3* in bronchial tissues; **(B)** quantification of pulmonary Sph and S1P by HPLC-MS/MS; **(C)** Western blot for ASAH1 in bronchial tissue; (**D** and **E**) *Spns2* expression analyzed by IHC (scale bar = 169.6 μm) and quantified by ImageJ/Fiji; and **(F)** qPCR analysis of *Spns2* in bronchial tissue. Data are expressed as means ± SEMs. Statistical analysis: unpaired *t* test. *P < .05, **P < .01.

Female mice exhibited significantly higher expression of *ORMDL3* and α-SMA in bronchial tissue compared to male animals, as demonstrated by IF analysis of pulmonary sections (Fig 2, E, F, and H) and qPCR analysis of isolated bronchi (Fig 2, G and I). To determine whether *ORMDL3* upregulation was associated with activation of the S1P signaling pathway, qPCR was performed on bronchial tissue for key enzymes involved in sphingolipid metabolism. Expression of sphingosine kinases SphK1 and SphK2, as well as S1P receptors S1PR1 and S1PR2, was significantly increased in bronchi from female mice compared to male animals (Fig 3, A). In contrast, expression levels of sphingomyelinase and S1PR3 did not differ between sexes (Fig 3, A). Analysis by HPLC-MS/MS revealed elevated pulmonary levels of both Sph and S1P in female compared to male mice (Fig 3, B). Consistent with these findings, Western blot analysis showed increased expression of acid ceramidase (ASAH1), the primary isoform involved in ceramide-to-Sph conversion and predominantly expressed in the bronchi, in female mice compared to male animals (Fig 3, C). Interestingly, this sex-based divergence in Sph and S1P levels was inversely correlated with the expression of the S1P transporter Spns2, which was upregulated in male mice, as evidenced by both IHC and qPCR analyses (Fig 3, D-F). These findings suggest a sex-different regulation of the ORMDL3-S1P axis in the airway, potentially contributing to differential airway responses.

### Targeting S1P signaling and estrogen receptors mitigates female-predominant asthmatic features in mice

**FIG 4.**
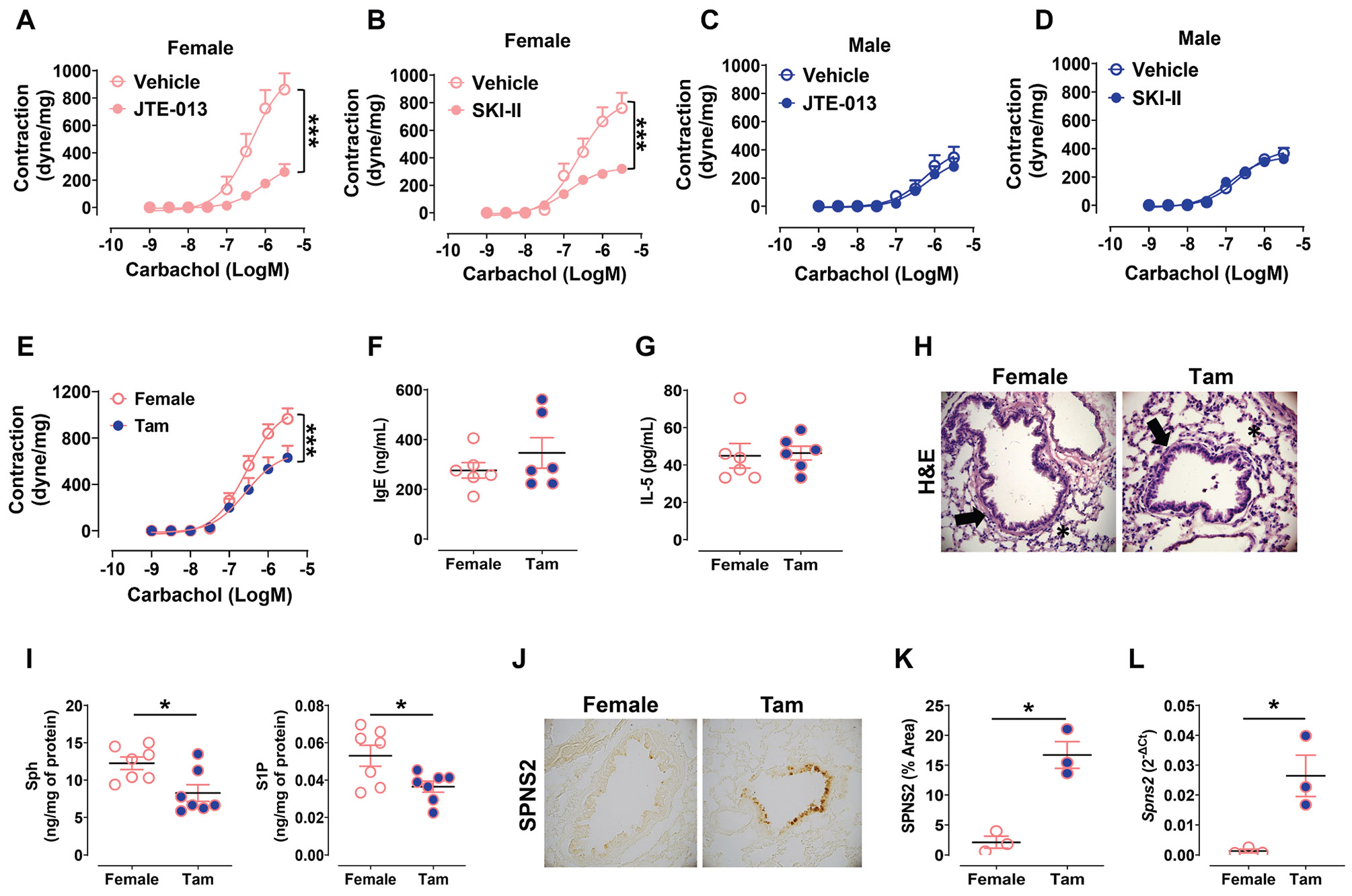
Sex-dependent airway responsiveness is mediated by sphingolipid signaling. **(A-D)** Bronchial tissues from male and female naïve BALB/c mice were exposed to cumulative carbachol in presence of either an S1PR2 antagonist (JTE-013, 10 μmol, 15 minutes) or a nonselective SphK inhibitor (SKI-II, 100 μmol, 1 hour). **(E-L)** Female mice received subcutaneously tamoxifen (Tam, 0.4 mg/kg per day for 7 days) or vehicle (Female); tissues were collected 7 days after first administration. *(E)* Airway reactivity. *(F)* Plasma IgE. *(G)* Pulmonary IL-5 levels. *(H)* Hematoxylin and eosin staining (scale bar = 169.6 μm). *(I)* Quantification of Sph and S1P by HPLC-MS/MS. (*J* and *K*) *Spns2* expression by IHC and ImageJ/Fiji quantification (scale bar = 169.6 μm). *(L)* qPCR analysis of bronchial *Spns2* expression. Data are expressed as means ± SEMs. Statistical analysis: 2-way ANOVA with Bonferroni posttest *(A-E)*, unpaired *t* test (*F, G, I, J*, and *L*). *P < .05, **P < .01, ***P < .001.

Pharmacologic inhibition of the S1P signaling pathway *in vitro* using JTE-013, a selective S1PR2 antagonist, or SKI-II, a SphK inhibitor, significantly attenuated bronchial hyperreactivity in female mice (Fig 4, A and B). In contrast, these treatments had no effect on bronchial reactivity in male mice (Fig 4, C and D), indicating a sex-specific dependence on S1P signaling. To further investigate the relationship between sex hormones and S1P-mediated airway reactivity, female mice were treated with tamoxifen (0.4 mg/kg per day for 1 week), a selective estrogen receptor modulator. Tamoxifen significantly reduced bronchial responsiveness to carbachol and eliminated the observed sex-related difference in airway reactivity (Fig 4, E). However, tamoxifen did not alter plasma IgE concentrations (Fig 4, F) or pulmonary IL-5 expression levels (Fig 4, G), suggesting its effects were not driven by systemic allergic sensitization or T_H_2 cytokine modulation.

Histologic analysis using hematoxylin and eosin staining demonstrated that tamoxifen treatment mitigated sex-specific structural alterations in the lung. Specifically, tamoxifen reduced the thickness of both the alveolar septa and the airway smooth muscle layer in female mice, aligning these parameters more closely with their male counterparts (Fig 4, H). In line with these morphologic changes, tamoxifen also decreased pulmonary levels of Sph and S1P in female mice (Fig 4, I). Sirius Red staining showed no significant sex differences in the overall extent of peribronchial or parenchymal collagen deposition (see Fig E2 in the Online Repository available at www.jacionline.org). However, confocal microscopy revealed a sex-dependent difference in the organization of peribronchial collagen fibers (Fig E2). In vehicle-treated female mice, peribronchial collagen appeared dense and highly organized, a pattern that was disrupted after tamoxifen treatment.

### Exogenous estrogen triggers asthma-like features in male mice by modulating *ORMDL3* expression and sphingolipid signaling

**FIG 5.**
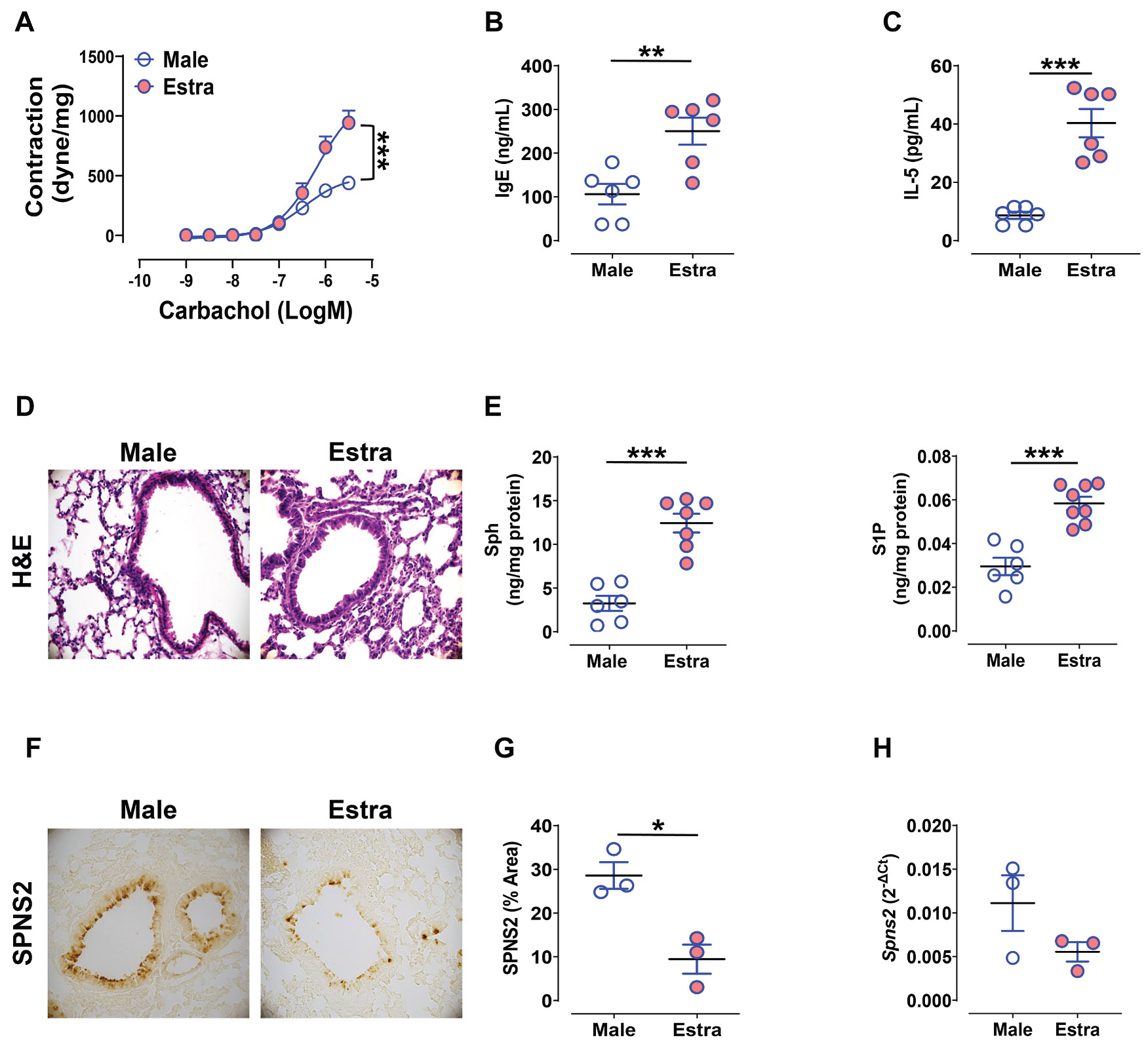
E2 supplementation in male mice abrogates sex differences in airway function and S1P pathway. Male BALB/c mice received E2 (25 μg/kg/d; Estra) or vehicle (Male) for 7 days. Tissues were collected on day 7 and analyzed for **(A)** airway reactivity and (**B** and **C**) plasma IgE and pulmonary IL-5 levels by ELISA. **(D)** Hematoxylin and eosin staining of lung sections (scale bar = 169.6 μm). **(E)** Pulmonary Sph and S1P levels by HPLC-MS/MS. (**F** and **G**) *Spns2* expression in lung tissue by IHC (scale bar = 169.6 μm) and ImageJ/Fiji analysis. **(H)** qPCR of bronchial *Spns2* expression. Data are expressed as means ± SEMs. Statistical analysis: 2-way ANOVA with Bonferroni posttest *(A)*, unpaired *t* test (*B, C, E, F*, and *H*). *P < .05, **P < .01, ***P < .001.

**FIG 6.**
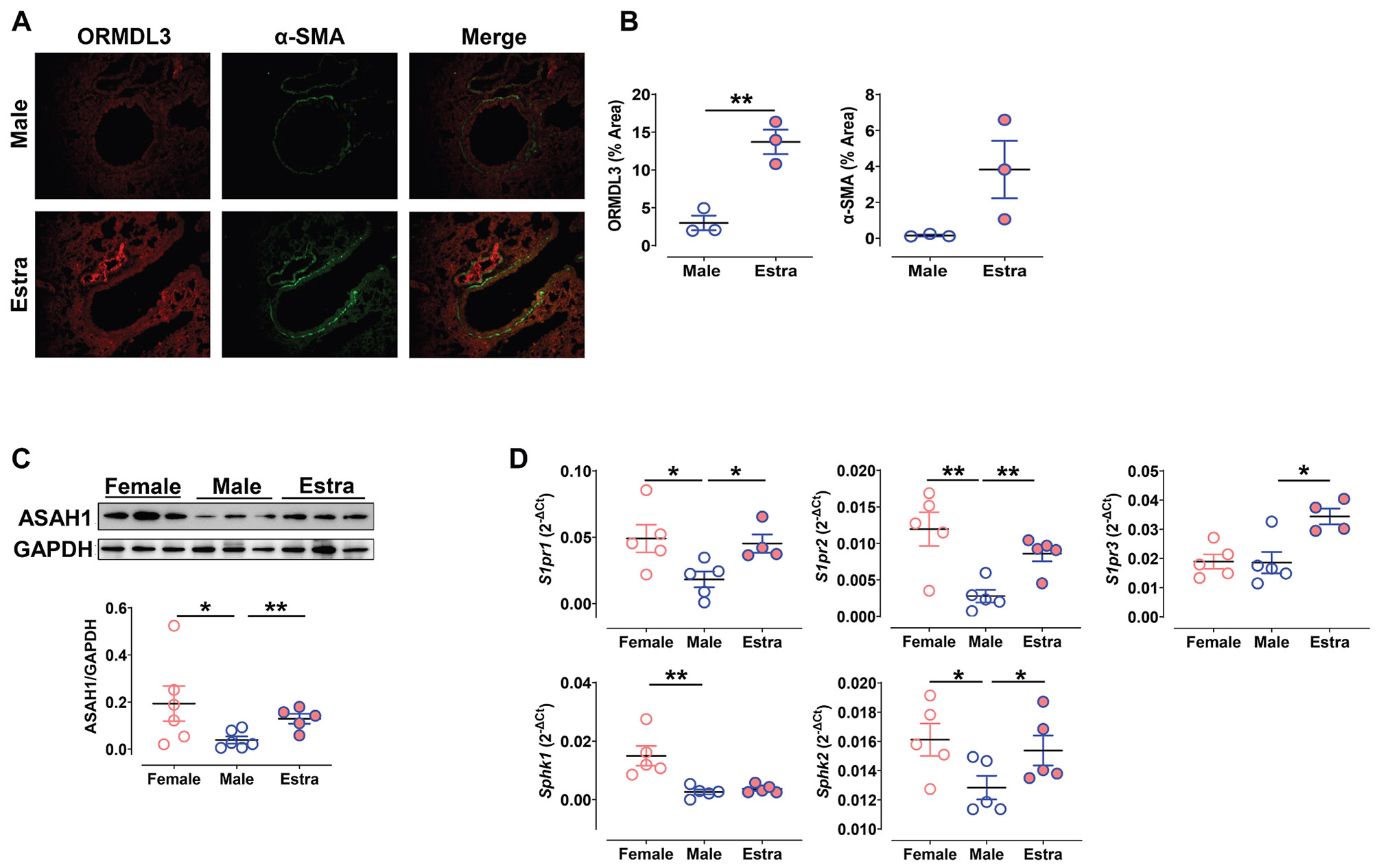
E2 enhances *ORMDL3* expression and S1P signaling in male mice. Male BALB/c mice were treated with E2 (25 μg/kg/d; Estra) or vehicle (Male) for 7 days and compared to female animals. (**A** and **B**) IF staining for ORMDL3 and α-SMA in lung sections (scale bar = 169.6 μm) and semiquantitative analysis via ImageJ/Fiji. **(C)** Western blot for ASAH1 in bronchi. **(D)** qPCR analysis of *S1pr1, S1pr2, S1pr3, Sphk1*, and *Sphk2* in bronchi. Data are expressed as means ± SEMs. Statistical analysis: unpaired *t* test *(B)* 1-way ANOVA (*C* and *D*). *P < .05, **P < .01.

Administration of E2 to male mice caused AHR (Fig 5, A) as well as increased plasma IgE (Fig 5, B) and pulmonary IL-5 (Fig 5, C) levels to the same levels observed in female mice. This effect was associated with an increase in both Sph and S1P lung levels (Fig 5, E). Accordingly, E2 reduced pulmonary *Spns2* expression to the values observed in female mice (Fig 5, F and G). These data were confirmed by qPCR (Fig 5, H). E2 also increased bronchial expression of ORMDL3 (Fig 6, A), α-SMA (Fig 6, B), and ASAH1 in male mice (Fig 6, C). qPCR performed on bronchi confirmed E2’s ability to upregulate all S1P receptors such as S1PR1, S1PR2, and S1PR3, as well as SPhk (Fig 6, D). Although E2 treatment in male animals did not affect both alveolar septa and smooth muscle layer, it influenced the organization of peribronchial collagen (Fig E2). E2 treatment in male mice makes the collagen structure more compact and organized, enhancing the collagen deposition not only in the peribronchial section but also in the lung parenchyma compared to vehicle-treated male animals (Fig E2).

### Allergen sensitization amplifies sex differences in airway remodeling and *ORMDL3* expression

**FIG 7.**
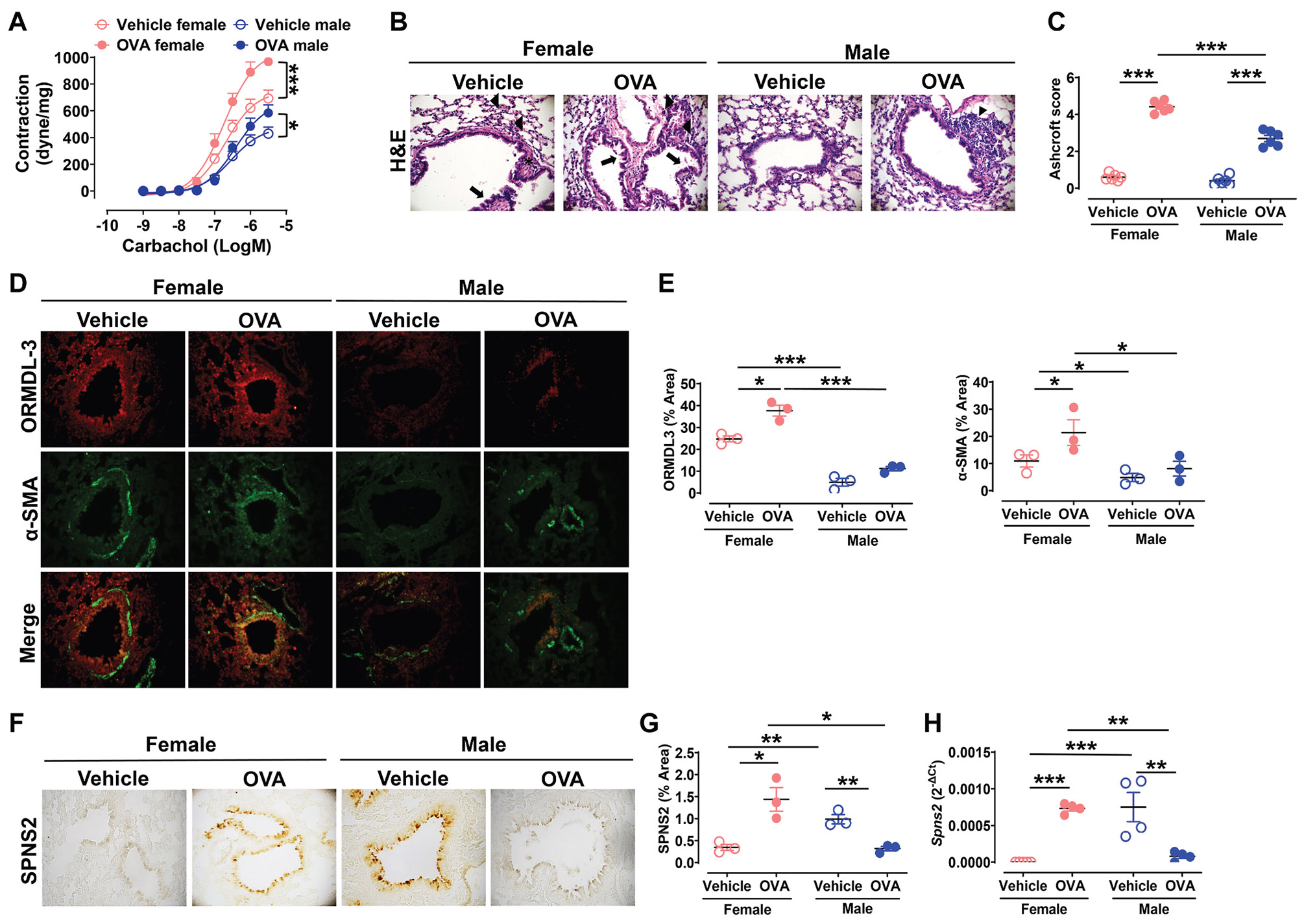
Allergen sensitization intensifies sex-based differences in airway responses and sphingolipid signaling. Male and female BALB/c mice were sensitized with OVA (100 μg on days 0 and 7). Tissues were collected on day 21 for: **(A)** airway reactivity in isolated bronchi; (**B** and **C**) hematoxylin and eosin staining of lung sections (scale bar = 169.6 μm) and Ashcroft score (0-5 scale for inflammation); (**D** and **E**) IF staining for ORMDL3 and α-SMA, and semiquantitative analysis (ImageJ/Fiji); and (**F** and **G**) IHC for SPNS2 (scale bar = 169.6 μm) and quantification by ImageJ/Fiji software. **(H)** qPCR of bronchial *Spns2*. Data are expressed as means ± SEMs. Statistical analysis: 2-way ANOVA with Bonferroni posttest *(A)*, unpaired *t* test (*C, E, G*, and *H*). *P < .05, **P < .01.

After OVA sensitization, both female and male mice exhibited a significant increase in bronchial reactivity; however, the sex-related difference in AHR persisted, with female mice displaying a greater response (Fig 7, A). This was accompanied by enhanced pulmonary inflammation in female compared to male mice, consistent with previous reports (Fig 7, B and C).^22^ Histologic analysis revealed sex-dependent structural differences in the lungs, even under baseline (vehicle) conditions. Specifically, female mice exhibited more pronounced alterations in the bronchial epithelium (Fig 7, B, *arrow*), parenchymal architecture (Fig 7, B*, arrowhead*), and peribronchial smooth muscle layer thickness (Fig 7, B*, asterisks*) compared to male mice. These structural differences were further exacerbated after OVA sensitization (Fig 7, C). OVA exposure also led to increased expression of *ORMDL3* and α-SMA in lung tissues from both sexes; however, the upregulation was significantly greater in female mice (Fig 7, D and E). In parallel, *Spns2* expression—implicated in S1P transport—was markedly elevated in bronchi isolated from sensitized female relative to male mice, as shown by IHC and qPCR (Fig 7, F-H). These findings reinforce a sex-difference in activation of the ORMDL3-S1P signaling axis in response to allergen exposure.

### Targeting S1P signaling mitigates asthma-like phenotypes specifically in female mice

**FIG 8.**
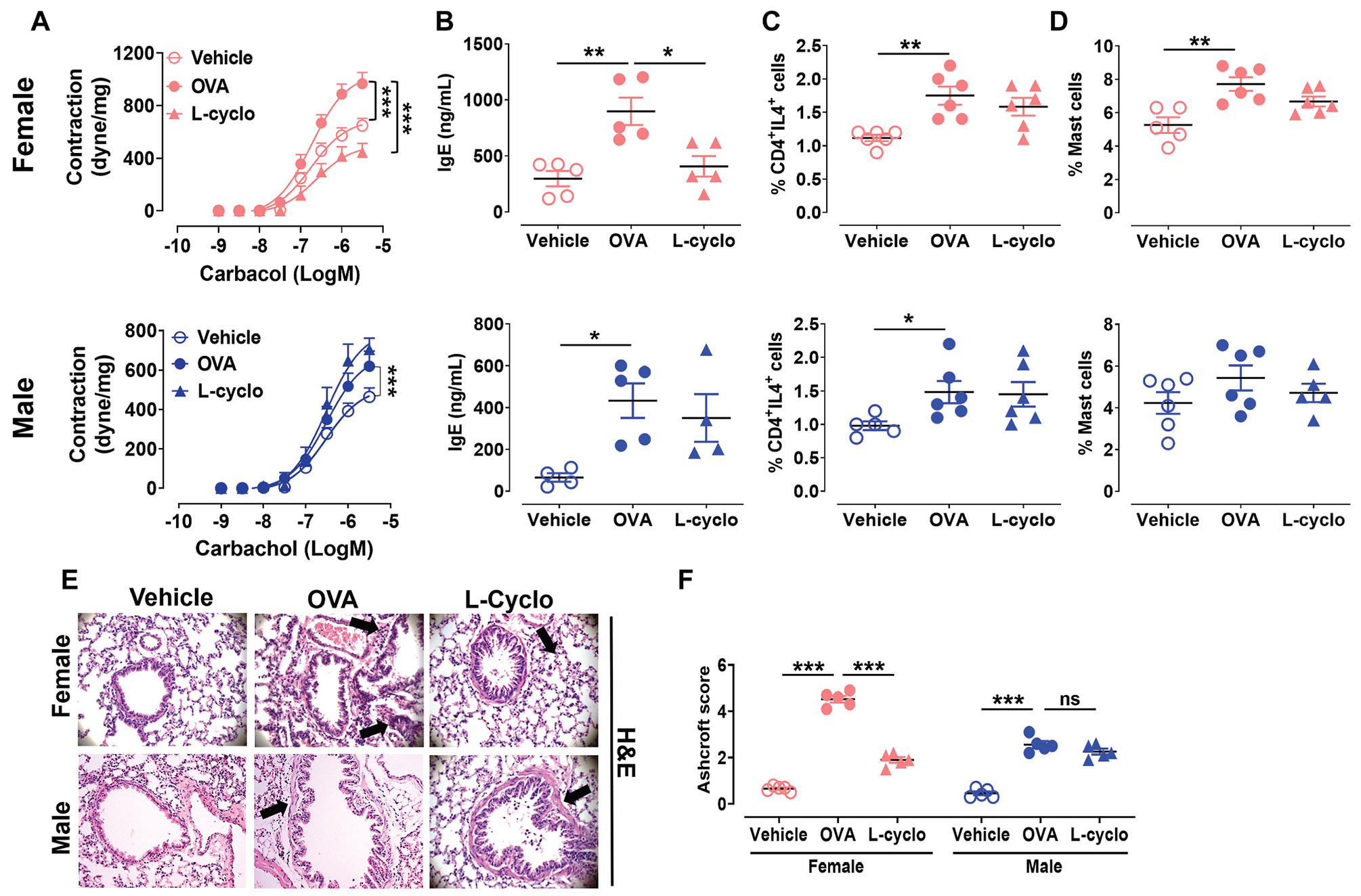
Pharmacologic inhibition of S1P signaling attenuates asthma features in a sex-dependent manner. Male and female OVA-sensitized BALB/c mice received l-cycloserine intraperitoneally 30 minutes before OVA. Tissues were analyzed for: **(A)** airway reactivity; **(B)** plasma IgE levels by ELISA; (**C** and **D**) flow cytometry for CD4^+^ IL4^+^ T cells and mast cells; **(E)** hematoxylin and eosin staining (scale bar = 69.6 μm); and **(F)** Ashcroft score (0-5 scale for inflammation). Data are expressed as means ± SEMs. Statistical analysis: 2-way ANOVA with Bonferroni posttest *(A)*, unpaired *t* test (*B, C, D*, and *F*). *P < .05, **P < .01, ***P < .001.

OVA-sensitized mice of both sexes were treated with l-cycloserine, an inhibitor of SPT to assess the impact of *de novo* sphingolipid biosynthesis inhibition on allergic airway inflammation. Functional assessments revealed that l-cycloserine significantly attenuated AHR in female mice, whereas no therapeutic effect was observed in male animals (Fig 8, A). In female mice, treatment also led to marked reductions in plasma IgE levels, pulmonary CD4 ^+^ T-cell infiltration, and mast cell accumulation (Fig 8, B-D). In contrast, these immunologic and functional parameters remained unchanged in male mice treated with l-cycloserine.

Histopathologic analyses further supported the sex-specific efficacy of l-cycloserine. In OVA-sensitized female mice, the treatment restored lung architecture, as evidenced by decreased alveolar septal thickening (Fig 8, E*, arrow*) and improved parenchymal organization (Fig 8, E and F). These structural improvements were not observed in their male counterparts, where smooth muscle thickness and parenchymal distortion persisted despite treatment (Fig 8, E*, arrow,* and F). Moreover, Picrosirius Red staining under polarized light microscopy (see Fig E3 in the Online Repository available at www.jacionline.org) revealed sex-dependent differences in peribronchial collagen organization after OVA sensitization. In female mice, l-cycloserine treatment effectively restored collagen fiber alignment and reduced aberrant deposition in both peribronchial and parenchymal regions. In male mice, however, collagen structure remained disorganized and unaffected by treatment.

## DISCUSSION

The role of sex hormones in asthma is highly complex, as reflected by heterogeneous biological and clinical features between sexes. Despite considerable progress, our mechanistic understanding of how sex hormones influence asthma phenotypes is incomplete. A deeper investigation into the biological underpinnings of sex differences in airway diseases remains critical. In this context, our study explores a novel potential role of ORMDL3 in modulating airway responses in a sex-specific manner, with a focus on its interaction with the sphingolipid biosynthetic pathway, a pathway already implicated in immune regulation and asthma pathogenesis.

The important role of ORMDL3 has been widely demonstrated by genetic studies, including genome-wide association studies, that have identified *ORMDL3* as a key susceptibility gene linked to asthma development and severity.^23^ Variants that increase *ORMDL3* expression correlate with heightened AHR, increased eosinophilic inflammation, and enhanced production of cytokines such as IL-4 and IL-13, all hallmarks of asthma.^24-26^ However, the precise role of ORMDL3 is context dependent, shaped by genetic background, environmental exposures, and age at onset.^23^ ORMDL3’s influence also varies by cell type, affecting airway epithelium and immune cells such as dendritic and innate lymphoid cells. These diverse effects position ORMDL3 as a key node linking susceptibility to asthma heterogeneity.

In this study, we examined the regulatory effects of sex hormones on *ORMDL3* expression and downstream sphingolipid signaling. The identification of ORMDL3 as a hormone-responsive modulator of airway function offers insight into how genetic susceptibility and hormonal signals intersect in asthma. Our findings provide compelling evidence that sex is a key determinant of airway responsiveness and remodeling, acting in part through modulation of the ORMDL3–sphingolipid signaling axis. *ORMDL3* expression was consistently higher in female lung tissue, both in human donors and murine models, and was associated with enhanced AHR, increased T_H_2-type inflammation, and structural airway remodeling. In human lungs, ORMDL3 levels positively correlated with the FEV_1_/FVC ratio, suggesting a link between epithelial *ORMDL3* expression and airway responsiveness.

Mechanistically, E2 emerged as a key upstream regulator of *ORMDL3* expression and sphingolipid metabolism. In BEAS-2B cells, E2 upregulated ORMDL3 and induced S1P signaling via transcriptional activation of key biosynthetic genes. Der p 1 also upregulated ORMDL3 but preferentially triggered inflammasome pathways. These distinct signaling cascades and differential effects on sphingolipid metabolism suggest that hormones like E2 can prime or reshape epithelial responses to allergens. Combination Der p 1/E2 produced a synergistic effect, amplifying *ORMDL3* expression and epithelial activation, suggesting that hormonal context can magnify allergen responses, especially in female subjects.

Sex hormones are critical modulators of lung structure and immunity.^14,27,28^ Estrogens increase lung volume but narrow airways, while androgens widen them. These divergent effects extend to immune responses, with female subjects typically showing stronger proinflammatory activity.^27,28^ In perfect tune in mouse models, female BALB/c mice exhibited stronger baseline and allergen-induced AHR, which correlated with greater ORMDL3, SphK, S1PR1/2 expression, and elevated S1P levels. Structural analyses also revealed sex-based differences in airway smooth muscle thickness, parenchymal architecture, and collagen organization—all more prominent in female subjects. Pharmacologic inhibition of S1P signaling via JTE-013, SKI-II, or tamoxifen reduced AHR and reversed remodeling in female subjects only, highlighting the sex-specific therapeutic potential of targeting this pathway.

To directly assess E2’s role, we administered E2 to male mice. This induced asthma-like features, with elevated AHR, IgE, and IL-5, mimicking the female phenotype. E2 also elevated pulmonary Sph and S1P levels and reduced *Spns2* expression, a transporter critical for S1P efflux,^11,29^ aligning expression patterns in male animals with those of female animals. ORMDL3 and downstream effectors were also upregulated, with notable collagen deposition despite the absence of allergen exposure. These data show that E2 alone can reprogram the male lung toward a female-like asthma phenotype, supporting hormone-driven regulation of ORMDL3 in sex-biased asthma.

To further dissect this axis, we used l-cycloserine,^30,31^ an inhibitor of SPT, to mimic ORMDL3 function. In OVA-sensitized mice, l-cycloserine selectively reduced AHR, inflammation, and remodeling in female animals only, restoring parenchymal and collagen architecture. This suggests that sex-specific metabolic programming contributes to disease pathogenesis.

In summary, our study identifies that sex differences in ORMDL-S1P signaling inform airway remodeling pathways, airway responsiveness, and responses to pathogen-associated stimuli such as Der p 1. However, the interplay of sex hormones, genetics, and environment is multifactorial and cannot be fully explained by a single pathway. Our conclusions are limited by the relatively small number of experiments conducted in human tissue. Future studies should prioritize validation in biospecimens from individuals with asthma, including both sexes, and incorporate detailed assessments of hormonal factors such as estrogen and testosterone. Direct analysis of ORMDL3 pathways in bronchial epithelial cells from well-characterized asthma patients will offer essential translational insight. Additionally, exploring ORMDL3’s roles in interferon and inflammasome pathways, particularly during viral infections, will expand our understanding of its function beyond type 2 inflammation. Recognizing sex as a critical biological variable remains essential for advancing personalized asthma therapies.

## METHODS

### Cell study

Male human bronchial epithelial cells (BEAS-2B) were obtained from ATCC and cultured in 75 cm^2^ flasks at a density of 1 × 10^6^ cells. Cells were maintained at 37°C in a humidified incubator with 5% CO_2_, and culture media were refreshed every 48 hours until 90% confluence was reached. For hormone stimulation, cells were treated with E2 (0.25 μmol; Sigma-Aldrich, E4389) or vehicle every other day for 5 days. Cells were subsequently exposed to *Dermatophagoides pteronyssinus* protein 1 (Der p 1, 10 ng/mL; MyBioSource, MBS485103) for 24 hours. At the conclusion of each treatment, cells were collected for qPCR analyses (Fig E1).

### *In vivo* study

Adult female and male BALB/c mice (8-9 weeks old) were obtained from Charles River Laboratories. Male mice received a daily subcutaneous injection of E2 (25 μg/kg; Sigma-Aldrich, E8875) or vehicle for 1 week. Female mice received a daily subcutaneous injection of tamoxifen, an estrogen receptor antagonist (0.4 mg/kg; Sigma-Aldrich, T5648), or vehicle for the same duration. Mice were humanely killed 7 days after the first injection. Lung slices obtained from male and female vehicle-treated BALB/c mice, tamoxifen-treated female animals, or E2-treated male animals were processed for Picrosirius Red staining. Lung slices were incubated with Picrosirius Red dye for 1 hour at room temperature and then rinsed twice in 8% acetic acid. Sirius Red staining was observed by bright-field microscopy for the analysis of collagen amount (Fig E2).

Male and female BALB/c mice (n = 6) received subcutaneous injections of chicken egg white OVA (100 μg, grade V, Sigma-Aldrich, A5503) emulsified in 3.3 mg of aluminum hydroxide gel (Al(OH)_3_; Supelco, 101091) in sterile saline (400 μL) on days 0 and 7. Control animals received an equivalent volume of sterile saline. Mice were humanely killed 21 days after the first OVA administration for sample collection. In a separate experimental group, mice were treated intraperitoneally with l-cycloserine, an SPT inhibitor (100 mg/kg; 100 μL), administered 30 minutes before each OVA injection. At the end point (day 21), all animals were anesthetized and humanely killed. Lung slices were incubated with Picrosirius Red dye for 1 hour at room temperature and rinsed twice in acetic acid 8%. Sirius Red staining was observed by bright-field microscopy for the analysis of collagen amount (Fig E3).

## Supplementary Material

1

## Key messages

- ORMDL3 shows sex-biased expression in airway tissues and contributes to differences in AHR and asthma-related features.
- E2 modulates *ORMDL3* expression and alters sphingolipid signaling pathways, supporting the existence of hormone-responsive asthma phenotypes.
- Understanding the interplay between sex hormones and sphingolipid metabolism may guide the development of sex-tailored therapeutic approaches in asthma.

## Abbreviations used

AHR: Airway hyperresponsiveness
α-SMA: Alpha smooth muscle actin
ASAH1: Acid ceramidase
E2: 17β-Estradiol
FEV _1_: Forced expiratory volume in 1 second
FVC: Forced vital capacity
HPLC-MS/MS: High-performance liquid chromatography coupled with tandem mass spectrometry
IF: Immunofluorescence
IHC: Immunohistochemistry
ORMDL3: Orosomucoid-like 3
OVA: Ovalbumin
qPCR: Real-time quantitative PCR
S1P: Sph-1-phosphate
Sph: Sphingosine
SphK: Sph kinase
SPT: Serine palmitoyltransferase
