# Supplementary material for: Sex hormones influence ORMDL3 expression: Implications for sex-associated asthma phenotype
Source: J Allergy Clin Immunol. Author manuscript; Available in PMC 2026 Jun 22. (PMC13285942; doi:10.1016/j.jaci.2025.10.035)
Supplement: 1 [file NIHMS2182968-supplement-1.pdf]

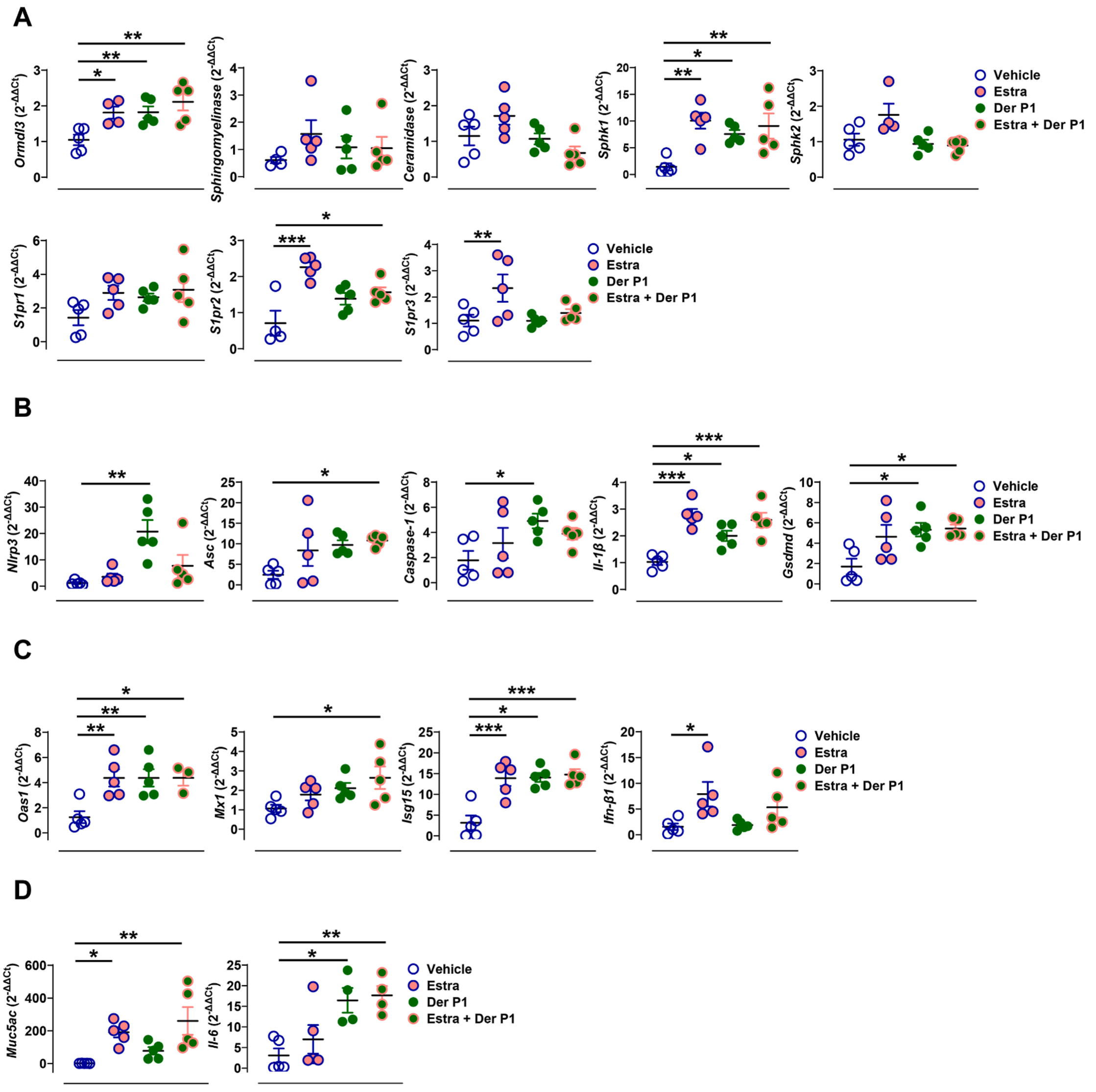

FIG E1. qPCR ($2^{-\Delta\Delta Ct}$) values for *ORMDL3, Ceramidase, Sphingomyelinase, SPHK1, SPHK2, S1PR1, S1PR2,* and *S1PR3* in BEAS-2B cells. Heat maps are shown of qPCR ($22^{-\Delta\Delta Ct}$) values for genes involved in inflammasome pathway *(NLRP3, ASC, Caspase-1, IL-1β, GSDMD)*, type I interferon pathway *(OAS1, MX1, ISG15, IFN-B1)*, and mucus/inflammatory markers *(MUC5AC, IL-6)*. Data are expressed as means ± SEMs. Statistical analysis: unpaired *t* test (*B* and *C*), 1-way ANOVA; $*P < .05$, $**P < .01$, $***P < .001$ vs vehicle.

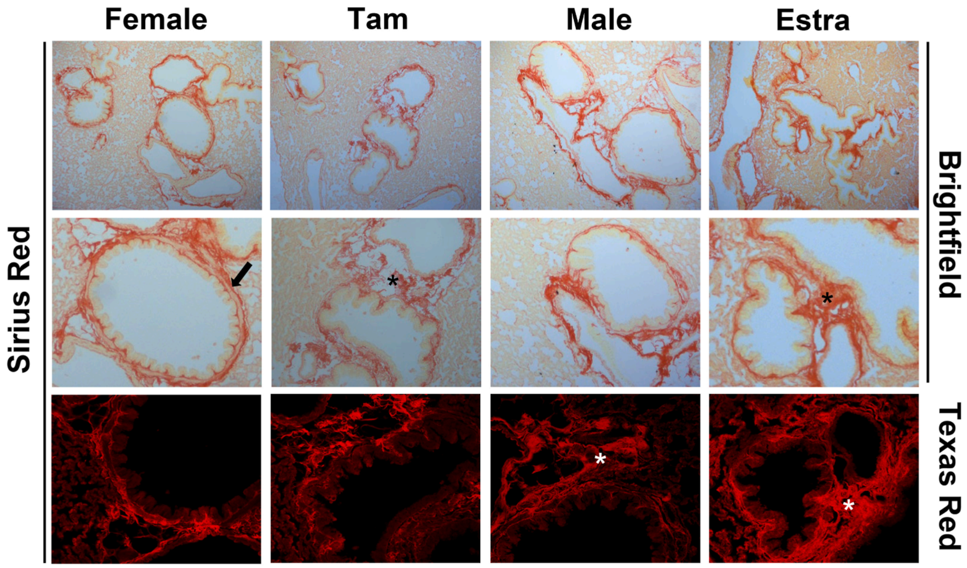

FIG E2. BALB/c received a subcutaneous injection of tamoxifen (0.4 mg/kg per day for 7 days) or vehicle every day for 1 week. Male BALB/c received subcutaneous injection of E2 (25 μg/kg) or vehicle every day for 1 week. Representative images of Picrosirius Red staining (scale bar = 169.6 μm and 85.6 μm for bright-field microscopy, and 85.6 μm for Texas Red); n = 6.

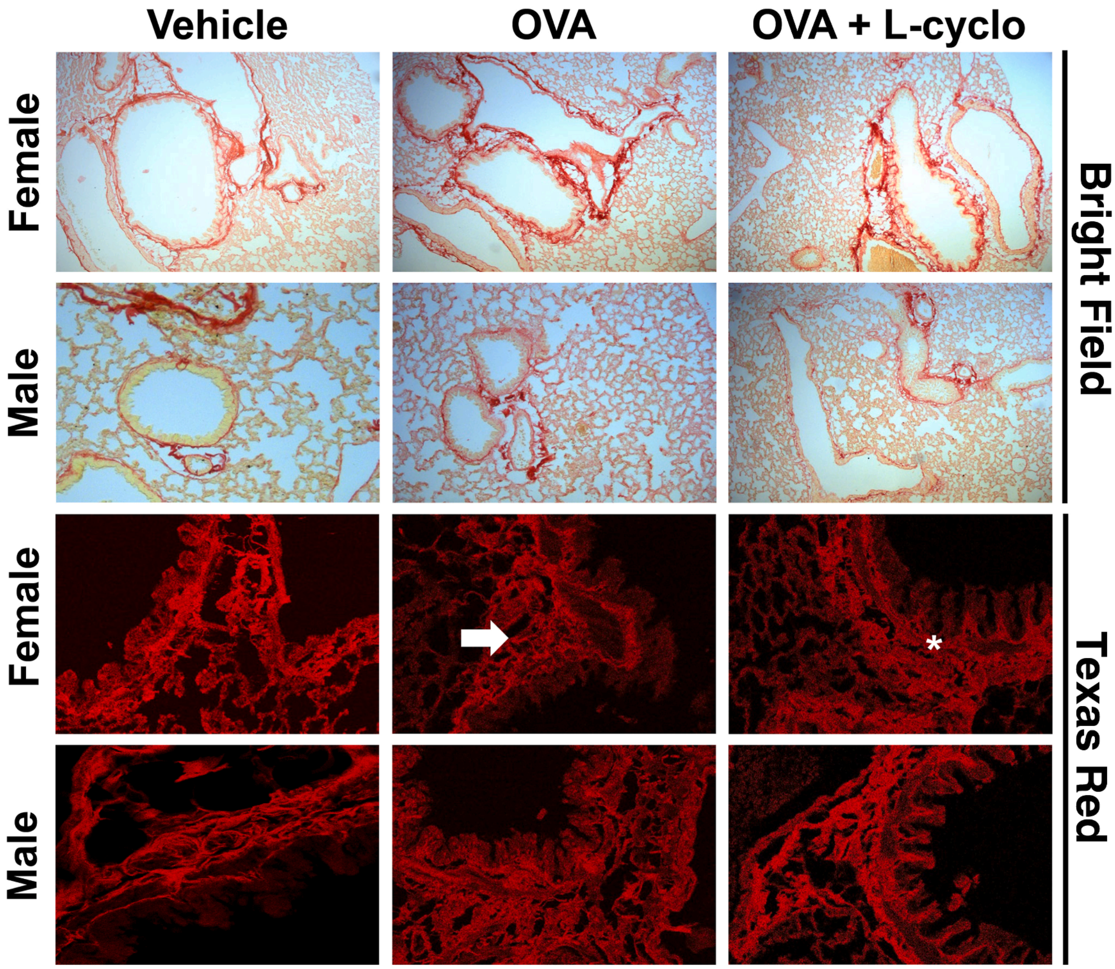

FIG E3. Male and female sensitized BALB/c mice received intraperitoneal L-cycloserine (L-cyclo) 30 minutes before OVA injection. Representative images of Picrosirius Red staining (scale bar = 169.6 μm and 85.6 μm for bright-field microscopy, and 85.6 μm for Texas Red); n = 6.

TABLE E1. Selected demographics, lung function, comorbidities, and medication

Characteristic	No.	Never-smoker control		*P*
		Male	Female	
No. of patients	9	5	4	
Age (years)	9	66.4	56	NS
% current smokers	9	0	0	NS
Race				
White	9	2	3	NS
Hispanic/Latino	9	3		NS
Black or African American	9		1	NS
Lung function				
%FEV_1	9	97.25	85	NS
FEV_1/FVC	9	74.33	85	.029*
Comorbidities				
Hypertension	9	3	0	NS
GERD	9	0	1	NS
Hyperlipidemia	9	2	1	NS
Diabetes mellitus	9	2	0	NS
Medications				
Antiplatelet	9	1	1	NS
Metformin	9	1	0	NS
ARB	9	1	1	NS
Insulin	9	1	0	NS

P values are derived from *t* test, applied for categorical variables like sex; *NS,* not statistically significant. *ARB,* angiotensin receptor blocker; *%FEV_1,* percentage FEV_1; *GERD,* gastroesophageal reflux disease.
*$P < .05$ male vs female subjects within same group.

TABLE E2. Lung function by sex

ID	Sex	$\%FEV_1$	FEV_1/FVC
1	F	94	86
2	F	74	85
3	F	87	84
4	F	NA	NA
5	M	85	68
6	M	97	NA
7	M	113	78
8	M	NA	NA
9	M	94	77

NA, Not available; *$\%FEV_1$*, percentage FEV_1.

TABLE E3. Primer sequences and genes screened by qPCR

Gene	Species	NCBI ID	Primer sequences (5′-3′)
S1pr1	Mouse	13609	Forward: CGCAGTTCTGAGAAGTCTCTGG<br>Reverse: GGATGTCACAGGTCTTCGCCTT
S1PR1	Human	1901	Forward: CCTGTGACATCCTCTTCAGAGC<br>Reverse: CACTTGCAGCAGGACATGATCC
S1pr2	Mouse	14739	Forward: TGTTGCTGGTCCTCAGACGCTA<br>Reverse: AGTGGGCTTTGTAGAGGACAGG
S1PR2	Human	9294	Forward: TGGAAACGCAGGAGACGACCTC<br>Reverse: CGAGTGGAACTTGCTGTTTCGG
S1pr3	Mouse	13610	Forward: GCTTCATCGTCTTGGAGAACCTG<br>Reverse: CAGAGAGCCAAGTTGCCGATGA
S1PR3	Human	1903	Forward: TTGTGGTGAGCGTGTTCATCGC<br>Reverse: AGCACAGCCAACACGATGAACC
Sphk1	Mouse	20698	Forward: GCTTCTGTGAACCACTATGCTGG<br>Reverse: ACTGAGCACAGAATAGAGCCGC
SPHK1	Human	8877	Forward: GCTGGCAGCTTCCTTGAACCAT<br>Reverse: GTGTGCAGAGACAGCAGGTTCA
Sphk2	Mouse	56632	Forward: GGTGCCAATGATCTCTGAAGCTG<br>Reverse: CTCCAGACACAGTGACAATGCC
SPHK2	Human	56848	Forward: GAGGAAGCTGTGAAGATGCCTG<br>Reverse: GAGCAGTTGAGCAACAGGTCGA
Asah1	Mouse	11886	Forward: GGATGTTCGGAAGGAAAGATGCC<br>Reverse: AACCCTCTCCAGACTTCTTGCC
ASAH1	Human	427	Forward: CTTTGCTGGCTATGTGGGCATG<br>Reverse: TGAGGAACCCTATCCACATGGC
Ormdl3	Mouse	66612	Forward: CATCTGGCTCTCCTACGTGCTG<br>Reverse: GTCCCTTTCACGGTGTGCAGAA
ORMDL3	Human	94103	Forward: GCAGGATGAATGTGGGCACA<br>Reverse: GGCAGGGGAAGGGGCTGCA
Gapdh	Mouse	14433	Forward: CATCACTGCCACCCAGAAGACTG<br>Reverse: ATGCCAGTGAGCTTCCCGTTCAG
GAPDH	Human	2597	Forward: CCACCCATGGCAAATTCCATGGCA<br>Reverse: TCTACACGGCAGGTCAGGTCCACC
